# Progress in Surgery

**Published:** 1900-02-24

**Authors:** 


### Progress in Surgery.

### SURGERY OF THE PERITONEUM.

**General Peritonitis** is almost beyond successful treatment, and our chances of success, by what are now commonly accepted methods of treatment, are universally proportional to the extent of peritoneum involved. Surgeons are apparently more or less agreed that it is best not to resort to general abdominal irrigation, if possible, and that in any case the cleansing of the peritoneum shall first be undertaken methodically from the point of infection outwards. If we can ever have an apparatus to supply hot salt solution continuously, and thus establish the same system that has been used in joints, we might save some otherwise hopeless cases of general peritoneal inflammation; but, until we can, the weight of evidence seems to be that there must be some provision for drainage.[1]

**Peritonitis Caused by the Lumbricus** is uncommon, but Katsurada[2] reports three cases—all fatal. In one of these—a girl aged 14—abdominal section was performed; no perforation was found, and a great quantity of purulent fluid was removed. The patient died on the second day. Between the spleen and the under surface of the diaphragm was a large round worm enclosed in an irregular cavity partly filled with dirty green pus, and partly traversed by newly-formed fibrous tissue. No trace of any perforation of the stomach or intestines could be found, but there were three round worms in the gastro-intestinal canal. There was no hepatic abscess, nor dilatation or obstruction of the ducts.

**Sub-Phrenic Abscess**, says Prescott le Breton,[3] is always secondary to traumatism or disease elsewhere. The most frequent cause is rupture of a gastric or duodenal ulcer; second in frequency is appendicitis, and other causes are abscess of the kidney, perinephritis, cholecystitis, traumatism to abdomen, rupture of typhoid ulcers of the intestine, echinococcus cyst of liver, pelvic abscess, pneumonia of lower lobe of right lung, and empyæma perforating the diaphragm. It is most often on the right side in the male from appendicitis and duodenal ulcer recurring more frequently in that sex, while it is more often left-sided in the female from the greater frequency of gastric ulcers. In about one-half the cases the sub-phrenic abscess contains gas from its communication with the gastro-intestinal tract. Leucocytosis is regularly present. Pain over the lower ribs radiating to the shoulder is a frequent symptom. There is a dome-shaped area of dulness corresponding to the convexity of the diaphragm; in empyæma, on the other hand, the line of dulness is concave upwards. Aspirated pus containing the bacillus coli communis indicates that the source of the pus is below the diaphragm. A sub-phrenic abscess lifts the diaphragm more than it depresses the liver, while abscess of the liver causes chiefly downward displacement of that organ. Treatment consists in resection of a part of the eighth, ninth, or tenth ribs in the middle axillary line, incision of the diaphragm, evacuation of the purulent contents, and drainage.

**The Radical Removal of Deep Pelvic Glands in Cases of Tuberculosis and Cancerous Infection** has been suggested by Lennander.[4] The operation he has devised is an extensive one, the external incision extending from the symphysis pubis along the course of Poupart's ligament to the anterior superior iliac spine, and then carried to about the middle of the crest of the ilium. From this incision a second one is carried downwards over the femoral vessels. In the course of the operation the inner attachment of Poupart's ligament is cut through, the epigastric and circumflex iliac arteries are divided, and the abdominal muscles attached to the crest of the ileum are incised to the full extent of the outer portion of the external wound. On stripping away the peritoneum, together with the vas deferens and spermatic cord, from the iliac fossa and the deep parts of the pelvis, both the common iliac artery and the external and internal iliac arteries are freely exposed. The vertical incision along the thigh affords access to the glands seated over the femoral vessels. This operation is held to be indicated by the following conditions: (1) In cases of cancer or sarcoma of the inguinal lymph glands, provided the primary growth can be radically removed, and the patient is free from other glandular metastases that cannot be effectually attacked. (2) In cases of extensive tuberculosis of the inguinal glands, in which a severe operation is not contra-indicated.

**Gun-shot Wounds of the Abdomen.**—Grant[5] states that the results of the investigations of Klemm showed, in an analysis of 152 cases, that even those patients in whom penetrating wounds of the abdomen were established, who did not die of immediate effects, eventually nearly all succumbed to exhaustion from sepsis, and later suppuration. This practically means that all cases of penetrating gun-shot wounds of the abdomen, not treated by surgical repair of the lesion, resulted fatally, from hæmorrhage, peritonitis, or sepsis. The practical management of suspected penetrating wounds is as plain a duty as the tying of a bleeding artery. As soon as possible after the injury, and under aseptic precautions, the experienced hand should carefully trace the course of the bullet into the cavity if it went there. The conclusion would seem that in all cases of gunshot wound of the abdomen, where the general circumstances are such as to warrant a major operation, the surgeon should open the abdomen at the earliest possible moment, effective exploration should be carried out, and any injury found dealt with as experience and the necessities of the moment would dictate.[6]

**Laparotomy for Uncontrollable Vomiting** was resorted to in a case brought forward by Ewart and Jaffrey.[7] The woman, æt. 49, had a pulsating swelling, supposed to be connected with the stomach, but this organ was found healthy though much dilated. An aneurism was found to be flattening the pyloric end of the stomach, but by bringing the stomach to the right the flattening was relieved. After operation vomiting ceased.

**Acute Fat Necrosis** terminating in recovery is recorded by McArthur.[8] The patient, a woman, was brought to the hospital in a state of profound collapse, with a history of having been seized with a sudden pain in the abdomen of intense character, resembling somewhat a hæmorrhage. The abdominal symptoms were sufficiently clear to conclude that fluid had escaped into the abdominal cavity. On opening the abdomen it was found that the cavity contained much straw-coloured fluid, and fluid was seen to be oozing from the lesser peritoneal pouch through a small opening in the omentum. The mesentery presented the condition recognised as acute fat necrosis, with sharply defined yellowish areas, but on putting the finger on it it will be recognised as being beneath the peritoneum. The finding of fluid in the lesser peritoneal pouch led at once to the thought of the emptying of a pseudo-cyst of the pancreas into this pouch, as not uncommonly happens, thus making the diagnosis clear. The lesser pouch was packed with a Mikulicz drain, the abdomen was washed out and drained. These areas of fat necrosis have been proved to be largely due to the combined action of pancreatic ferment and bacteria, the bacillus coli communis being found there.

[1] Med. Rec., Dec. 23, 1899, p. 933. [2] Brit. Med. Jour., Oct. 29, 1899, Epitome, No. 331; and Sei-i-Kwai Med. Jour., April 30, 1899. [3] Brit. Med. Jour., Dec. 30, 1899, Epitome No. 492; and Buffalo Med. Jour., lv., 255. [4] Brit. Med. Jour., Jan. 6, 1900, Epitome No. 5; and Centralbl. für Chir., No. 37, 1899. [5] Med. Rec., Dec. 16, 1899, p. 906. [6] Brit. Med. Jour., Oct. 28, 1899, p. 1210. [7] Ibidem, p. 1198. [8] New York Med. Jour., Nov. 4, 1899, p. 684.

### GENERAL SURGERY.

**Antiseptics.**—Scheller[1] has carried out experiments comparing europhen and iodoform in the treatment of suppurating wounds, and has arrived at the following conclusions. Eczema is never observed after the employment of europhen, nor irritating symptoms of any kind. Healing took place quickly, and the comparatively slight scar formation was remarkable. The disagreeable odour from purulent surfaces, which is but little improved by iodoform, is also considerably reduced by europhen. Owing to the rapid splitting up of iodine, europhen prevents sepsis *in status nascendi*, and to a much greater extent than iodoform. The pain allaying and antiseptic action of europhen makes it also a valuable remedy in burns, abscesses, chilblains, furuncles, &c. Shield[2] relates a case of acute mania terminating in death on the fourth day after removal of the mamma, probably due to iodoform poisoning. Pridgin Teale has experienced similar symptoms, uncontrollable delirium and high temperature, in breast cases after the use of iodoform. Shield has known of two fatal cases which have not been reported in London practice in the last two years. In the one iodoform was used in the amputation, with a fatal result certainly attributable to the drug. Certain patients are peculiarly liable to iodoform poisoning. In most cases the drug may be used freely and nothing happen; however, the danger is a real one. In sloughing wounds or foul intestinal abscesses, Shield does not hesitate to use iodoform freely, but in fresh wounds and about freshly made incisions he considers it better to avoid its use. Noguera,[3] in discussing the use of xeroform in military surgery considers that it is very valuable when large numbers of wounded have accumulated, since it dried up the moisture secreted from the exposed surfaces and sterilised them. He is convinced that a simple xeroform dry dressing applied on the battlefield would keep the wounds aseptic for two or three days, thereby giving more than sufficient time for removal of the patients to the hospitals. A new antiseptic ointment for surgical use named Lyptol[4] has lately been introduced. It is composed of a sterilised petroleum base to which hyd. bichlor. oleum eucalypt. formalin, and benzo-boracic acid are added. Lyptol is claimed to be eminently antiseptic and germicidal, and to have the power to prevent putrefactive decomposition of organic material as well as the development of bacteria of any kind. It is especially useful in venereal sores, varicose ulcers, affections of the mucous membranes, and pruritus vulvæ and ani. Of all the circumstances influencing the progress of surgery in the past quarter of a century the development of the antiseptic theory and practice had been undoubtedly the most powerful. Sir William Savory, according to H. C. Clark,[5] published, in the early days of the antiseptic controversy, statistics to show that his results under old methods were as good as those of the most advanced advocates of antisepticism. He expressed the opinion that cases of strangulated hernia should be excluded from such statistics, on the ground that the recovery of the patient depended entirely on the condition of the bowel and not on the kind of dressing used. Although there was much truth in this, the statistics of strangulated hernia show a progressive diminution of mortality under antiseptic treatment, and it must be apparent that a wound which heals without suppuration must result in a more rapid cure, cause less risk to the patient's life, and give a better cicatrix than when the wound is septic. In a recent article Carl Beck[6] gives a brief *résumé* of some points regarding the perfection of asepsis. One important one is as follows. As he truly states, the atmosphere contains an enormous number of bacteria, but these being innocent mould, yeast, and fission fungi, they are fortunately non-pathogenic for the human race. Bacteria which are pathogenic for man are present in the atmosphere only under abnormal conditions, viz., when they are stirred up from their natural habitat, the earth's surface, or the dust of the walls, the floor, the tables, &c. The properties of the atmosphere are, in fact, injurious to pathogenic bacteria in every respect. As regards the skin of the patient, although its surface can be rendered sterile by the usual methods, yet underneath the skin surface a number of bacteria are sheltered by the glands of the skin, the secretion of which offers a favourable soil for their development. These are not accessible to any disinfection or removal. In incising the skin the knife divides a number of these glands and exposes the bacteria. This explains the so-called stitch suppurations and the numerous incomprehensible infections which occur with extremely careful aseptic surgeons. Not only the knife but the surgeon's hands are necessarily infected by the intimate contact with these deep-skin bacteria. This indicates the change of the infected knife, and the redisinfection of the surgeon's hands. The latter procedure may become unnecessary if gloves are worn by the surgeon while the skin is being incised.

[1] The Therapist, May 15, 1899: and Pharmaceut. Centralblatt, No. 12, 1899. [2] The Clin. Jour., Oct. 4, 1899, p. 382. [3] Lancet, Nov. 25, 1899; and Revista de Medicina of Chirurg. Practicas (Madrid), April 25, 1899. [4] Pamphlet, Thos. Chirty and Co., 1899. [5] Glasgow Med. Jour., Nov., 1899, p. 307. [6] Med. Rec., New York, Oct. 7, 1899, p. 305.

